# Atypical homodimerization revealed by the structure of the (*S*)-enantioselective haloalkane dehalogenase DmmarA from *Mycobacterium marinum*

**DOI:** 10.1107/S2059798323006642

**Published:** 2023-10-20

**Authors:** Karolina Snajdarova, Sérgio M. Marques, Jiri Damborsky, David Bednar, Martin Marek

**Affiliations:** a Loschmidt Laboratories, Department of Experimental Biology and RECETOX, Faculty of Science, Masaryk University, Kamenice 5, Building A13, 625 00 Brno, Czech Republic; b International Clinical Research Center, St Anne’s University Hospital Brno, Pekarska 53, 656 91 Brno, Czech Republic; Station Biologique de Roscoff, France

**Keywords:** haloalkane dehalogenases, *Mycobacterium marinum*, DmmarA, homodimerization, surface loops, enantioselectivity, X-ray crystallography, SAXS

## Abstract

Haloalkane dehalogenases (HLDs) are a family of α/β-hydrolase fold enzymes that employ S_N_2 nucleophilic substitution to cleave the carbon–halogen bond in diverse chemical structures, the biological role of which is still poorly understood. Atomic-level knowledge of both the inner organization and supramolecular complexation of HLDs is thus crucial to understand their catalytic and noncatalytic functions. Here, crystallographic structures of the (*S*)-enantio­selective haloalkane dehalogenase DmmarA from the waterborne pathogenic microbe *Mycobacterium marinum* were determined at 1.6 and 1.85 Å resolution. The structures show a canonical αβα-sandwich HLD fold with several unusual structural features. Mechanistically, the atypical composition of the proton-relay catalytic triad (aspartate–histidine–aspartate) and uncommon active-site pocket reveal the molecular specificities of a catalytic apparatus that exhibits a rare (*S*)-enantiopreference. Additionally, the structures reveal a previously unobserved mode of symmetric homodimerization, which is predominantly mediated through unusual L5-to-L5 loop interactions. This homodimeric association in solution is confirmed experimentally by data obtained from small-angle X-ray scattering. Utilizing the newly determined structures of DmmarA, molecular modelling techniques were employed to elucidate the underlying mechanism behind its uncommon enantioselectivity. The (*S*)-preference can be attributed to the presence of a distinct binding pocket and variance in the activation barrier for nucleophilic substitution.

## Abstract 2

Crystallographic structures of the (*S*)-enantioselective haloalkane dehalogenase DmmarA from the waterborne pathogenic microbe *Mycobacterium marinum* were determined at 1.6 and 1.85 Å resolution. The structures reveal a previously unobserved mode of homodimerization, which is predominantly mediated through unusual L5-to-L5 loop interactions.

## 1. Introduction

Haloalkane dehalogenases (HLDs) are α/β-hydrolase fold enzymes that catalyse the cleavage of carbon–halogen bonds in halogenated compounds through an S_N_2 nucleophilic substitution mechanism, producing a corresponding alcohol, a halide and a proton (Verschueren *et al.*, 1993 ▸). Their most important biotechnological applications include (i) bio­remediation, including, for instance, biodegradation of pollutants such as 1,2-dichloroethane or 1,2,3-trichloropropane, (ii) decontamination of the warfare agent yperite, (iii) pollutant biosensing and (iv) cell imaging as represented by the HaloTag technology (Koudelakova *et al.*, 2013 ▸; Marques *et al.*, 2022 ▸). The HLD family is divided into three subfamilies (HLD-I, HLD-II and HLD-III) based on sequence and phylo­genetic analyses of the whole HLD family (Chovancová *et al.*, 2007 ▸).

Recently, database-mining searches identified a new haloalkane dehalogenase, DmmarA, encoded in the genome of the waterborne pathogenic bacterium *Mycobacterium marinum* M. This bacterium causes a tuberculosis-like illness in fish and can lead to infections in humans, particularly those with immune deficiencies (Akram & Aboobacker, 2022 ▸). DmmarA belongs to the HLD-II subfamily. This subfamily has a catalytic pentad consisting of two halide-stabilizing residues, asparagine–tryptophan, and a proton-relay system composed of the catalytic triad aspartate–histidine–glutamate (Chovancová *et al.*, 2007 ▸). However, DmmarA differs from the other members of the HLD-II subfamily as it contains an aspartic acid instead of a glutamic acid as the catalytic acid in the catalytic triad. Therefore, its catalytic pentad consists of the noncanonical triad aspartate–histidine–aspartate and two canonical residues, asparagine and tryptophan, for halide stabilization. From previous high-throughput characterization experiments, we learnt that DmmarA forms a dimeric structure and exhibits an (*S*)-enantiopreference (namely with 2-bromopentane), which is rare across the whole HLD family (Vasina *et al.*, 2022 ▸).

The molecular structures of HLDs in both the HLD-I and HLD-II subfamilies are mostly monomeric (Kunka *et al.*, 2018 ▸), with the exception of a few proteins as listed below. The proteins in the HLD-III subfamily form highly polydisperse supramolecular complexes (Jesenská *et al.*, 2009 ▸) and no structures have been experimentally determined to date. Oligomeric forms of proteins from the HLD-I and HLD-II subfamilies have been reported in nine crystal structures deposited in the Protein Data Bank (PDB; Table 1 ▸). Six of these nine proteins also exist as oligomers in solution (DpaA, DatA, DbeA, DmmA, DmxA and DbjA). On the other hand, DppA and HanR are considered to be monomeric in solution (Hesseler *et al.*, 2011 ▸; Novak *et al.*, 2014 ▸) and their oligomeric nature was only the result of crystal packing. The oligomeric form of DccA in solution has not been experimentally confirmed (Carlucci *et al.*, 2016 ▸).

Here, we attempted to crystallize the haloalkane dehalogenase DmmarA from *M. marinum* M. Diffraction-quality crystals were grown under chemically distinct conditions, and the corresponding structures were determined at 1.6 and 1.85 Å resolution by X-ray crystallography. Mechanistically, the atypical composition of the catalytic triad (aspartate–histidine–aspartate) and the unique constellation of residues in the active-site pocket reveal the molecular specificities of a catalytic apparatus that exhibits the rare (*S*)-enantio­preference of this enzyme family. Moreover, the structures reveal an previously unobserved mode of symmetric homodimerization mediated through unusual L5-to-L5 loop interactions. Our findings thus highlight the key molecular features that distinguish the DmmarA enzyme from other HLD family members. They provide a structural basis for the design of inhibitors to impair the pathogenic microbe by targeting its dehalogenation modifications.

## 2. Materials and methods

### 2.1. Gene synthesis and protein overproduction

The pET-24a plasmid containing the *dmmarA* gene was synthesized commercially (BaseClear B.V., The Netherlands). The construct encompasses NdeI and XhoI restriction sites, kanamycin resistance and a hexahistidine tag at the C-terminal end. The pET-24a-DmmarA plasmid carrying the *dmmarA* gene was transformed into chemocompetent *Escherichia coli* BL21 (DE3) cells using the heat-shock method (30 min on ice, 50 s at 42°C and 3 min on ice). 200 µl SOC (Super Optimal Broth with catabolite repression) medium was added and the cells were incubated at 37°C for 1 h and then cultivated on LB–agar plates with kanamycin (35 µg ml^−1^) at 37°C overnight. The next day, pre-cultures were prepared by transferring several colonies into 10 ml LB medium with kanamycin (35 µg ml^−1^). The pre-cultures were cultivated at 37°C and 200 rev min^−1^ for 3 h. Each pre-culture was used to inoculate 1 l LB medium with kanamycin (35 µg ml^−1^) and was cultivated at 37°C and 130 rev min^−1^ until the optical density (OD_600_) reached 0.4–0.6. Gene expression was induced by the addition of isopropyl β-d-1-thiogalactopyranoside (IPTG) to a final concentration of 200 µ*M* (0.5 ml 1 *M* IPTG in 1 l LB medium). The cells were incubated at 20°C and 130 rev min^−1^ overnight. On day 3, the cells were harvested by centrifugation (4°C and 4000 rev min^−1^ for 10 min). The supernatant was discarded and the pellet from each 2 l LB medium was resuspended in 30 ml buffer (10 m*M* Tris, 50 m*M* sodium formate, 10 m*M* imidazole pH 8.5). The harvested cell mass was stored at −70°C.

### 2.2. Protein purification by two-step chromatography

The suspension with the harvested cells was defrosted and 90 µl of DNAse was added (∼2 µg ml^−1^). The cell suspension was sonicated using a Fisherbrand Model 705 Sonic Dismembrator in three 2 min cycles. The disrupted cells were centrifuged (4°C and 14 000 rev min^−1^ for 1 h). The protein was then filtered and loaded onto a BioLogic DuoFlow FPLC system (Bio-Rad, USA) equilibrated in buffer *A* (10 m*M* Tris, 50 m*M* sodium formate, 10 m*M* imidazole pH 8.5) and buffer *B* (10 m*M* Tris, 50 m*M* sodium formate, 500 m*M* imidazole pH 8.5). The protein was purified by metal-affinity chromatography using a nickel-charged column at a flow rate of 1 ml min^−1^. The protein was eluted with an increasing gradient of imidazole (0%, 10%, 60% and 100% buffer *B*; DmmarA was eluted in the 10% and 60% gradients). The purified protein from the 60% imidazole gradient was concentrated to ∼5 ml using Amicon Ultra-15 Centrifugal Filter Units (10 kDa cutoff).

5 ml of protein was loaded onto an ÄKTApure FPLC system (Cytiva, Sweden) equilibrated in gel-filtration buffer (10 m*M* Tris, 50 m*M* sodium formate pH 8.5). The protein was purified by size-exclusion chromatography using a Superdex 16/60 200 pg column (GE Healthcare, UK). SDS–PAGE was used to check the purity of fractions from affinity chromatography and size-exclusion chromatography (run at 200 V and 400 mA for 40 min).

### 2.3. Differential scanning fluorimetry

The purified protein at a concentration of 0.48 mg ml^−1^ was used for protein stability measurements. The thermal unfolding of the enzyme was analysed by differential scanning fluorimetry on a NanoTemper Prometheus NT.48 in three capillaries. The experiment was performed at temperatures ranging from 20 to 98°C. The melting temperature was deduced from the ratio of tryptophan fluorescence at 350 and 330 nm.

### 2.4. Crystallization experiments

The protein was concentrated to ∼10 mg ml^−1^ for crystallographic experiments. Initially, crystallization screenings were performed using a Gryphon LCP crystallization robot (Art Robbins Instruments, USA) in 96-well plates (SWISSCI, Switzerland) using the sitting-drop vapour-diffusion technique (1:1 or 1:2 precipitant:protein ratio) at 20°C. Several commercial screens were used in the experiment.

After one week, crystal growth was monitored under the microscope and plausible conditions were further tested at 18°C in 15-well plates (EasyXtal, USA) using the hanging-drop vapour-diffusion technique (1:1, 1:1.5 or 1:2 precipitant:protein ratios with 500 µl precipitant solution in the reservoir). Different PEG concentrations were tested in this screening. After one week, the best crystallization conditions were chosen. Crystals were found in two solutions, the first composed of 0.02 *M* KH_2_PO_4_, 0.1 *M* bis-Tris propane pH 6.5, 22% PEG 3350 and the second composed of 0.2 *M* ammonium acetate, 0.1 *M* bis-Tris pH 5.5, 20% PEG 3350. The crystals obtained in these conditions were cooled in liquid nitrogen in cryosolutions corresponding to the crystallization solutions supplemented with 20% glycerol.

### 2.5. Data collection and structure refinement

X-ray diffraction data were collected on the PXIII beamline at the SLS synchrotron at a wavelength of 1.0 Å. The collected diffraction images were processed using the *XDS* software (Kabsch, 2010 ▸) and the data were reduced in *AIMLESS* (Evans & Murshudov, 2013 ▸). The contents of the asymmetric unit were estimated by calculating the Matthews coefficient (Weichenberger & Rupp, 2014 ▸). Molecular replacement was performed by *Phaser* (McCoy *et al.*, 2007 ▸) in the *Phenix* software suite (Liebschner *et al.*, 2019 ▸) using a DmmarA model built by *SWISS-MODEL* (Waterhouse *et al.*, 2018 ▸) based on its 53.57% sequence similarity to the haloalkane dehalogenase LinB (PDB entry 4wdq; Brezovsky *et al.*, 2016 ▸). Several cycles of automatic refinement were carried out in *Phenix* and the structures were manually refined in *Coot* (Emsley *et al.*, 2010 ▸). Ligands were built using *eLBOW* (Moriarty *et al.*, 2009 ▸). The structures of HLD dimers were superimposed with the *SSM superpose* function in *Coot* (Emsley *et al.*, 2010 ▸). The final structures were visualized in *PyMOL* (version 2.0; Schrödinger).

### 2.6. Protein characterization using structural bioinformatics tools

The protein interfaces were analysed by the *PDBePISA* server (Krissinel & Henrick, 2007 ▸) and noncovalent inter­actions between chains were evaluated using the *PLIP* web tool (Adasme *et al.*, 2021 ▸). A multiple sequence alignment of similar HLDs was created using the *MAFFT* server (Katoh *et al.*, 2019 ▸), and the sequence and secondary-structure similarities were rendered by *ESPript* (Robert & Gouet, 2014 ▸). HLD protein structures were compared pairwise using the *DALI* server (Holm, 2020 ▸). *AlphaFold2* (Mirdita *et al.*, 2022 ▸) was used to predict the conformation and assembly of DmmarA.

### 2.7. Small-angle X-ray scattering (SAXS)

The oligomeric state of DmmarA in solution was determined by SAXS. The SAXS data were collected in the Rigaku BioSAXS-1000 chamber at CEITEC (Brno, Czech Republic). The protein was purified by size-exclusion chromatography on an ÄKTApure FPLC system (Cytiva, Sweden) equilibrated in 50 m*M* sodium acetate, 10 m*M* Tris pH 8.5 and equipped with a HiLoad 16/600 Superdex 200 prep-grade column (GE Healthcare, UK). The SAXS data were measured using three different concentrations of the enzyme: 2, 4.5 and 8.7 mg ml^−1^. The buffer from size-exclusion chromatography (50 m*M* sodium acetate, 10 m*M* Tris pH 8.5) was used as a blank. The scattering curves were fitted to the crystallographic monomer and dimer structures using *CRYSOL* from *ATSAS* v.2.8.4 (Svergun *et al.*, 1995 ▸). *Ab initio* modelling was performed by *DAMMIN* from *ATSAS* (Svergun, 1999 ▸). The SAXS *ab initio* model was superposed with the crystallographic structure of DmmarA in *SUPCOMB* from *ATSAS* (Kozin & Svergun, 2001 ▸) and visualized in *PyMOL*.

### 2.8. Molecular docking

The structures of the ligands [the (*R*)- and (*S*)-enantiomers of 2-bromopentane and 2-bromohexane] were constructed in *Avogadro* 1.2.0 (Hanwell *et al.*, 2012 ▸) and minimized using the Universal Force Field (UFF; Rappe *et al.*, 1992 ▸) and the steepest-descent algorithm. The semi-empirical AM1-BCC function (Jakalian *et al.*, 2000 ▸, 2002 ▸) was employed to calculate the partial atomic charges of the ligands using the *antechamber* module of *AmberTools* 14 (Case *et al.*, 2014 ▸). The three-dimensional structures of the proposed fluorescent compounds were prepared in *Avogadro* and were minimized using the UFF force field and the steepest-descent algorithm. The three-dimensional structures of the receptors were obtained from the RCSB Protein Data Bank (Berman *et al.*, 2000 ▸) for DbjA (PDB entry 3a2m; Prokop *et al.*, 2010 ▸) or from this work after solving the crystallographic structure (DmmarA). Only the first chain of each structure was used, and the chains were aligned with *PyMOL* (version 1.7.4; DeLano, 2002 ▸). Water molecules, ions and co-crystallization molecules were removed. The double side chains of several residues were also removed, leaving only the conformations that were more frequently observed among all of the structures. H atoms were added to structures that lacked them with the *Reduce* program of *AmberTools* 14 (Case *et al.*, 2014 ▸) using dynamic optimization of their position (-build -nuclear options). The input files of the ligands and receptors in PDB format were converted to the *AutoDock Vina*-compatible format PDBQT using *MGLTools* (Sanner, 1999 ▸), maintaining the previously calculated atomic charges for the ligands. The active site of the haloalkane dehalogenases was selected as the region of interest for molecular docking performed by *AutoDock Vina* 1.1.2 (Trott & Olson, 2010 ▸). This region was represented by a box of 20 × 20 × 20 Å centred at the coordinates of the CG atom of the catalytic residue Asp95 of DmmarA. The exhaustiveness parameter was defined as 100 to increase the conformational search, the energy range was increased to 10 kcal mol^−1^ to obtain a higher number of binding poses and the maximum number of modes saved was increased to 20. The docked poses were rescored using the Smina scoring function (Koes *et al.*, 2013 ▸).

All of the docking binding modes were analysed using *PyMOL* version 1.7.4. The identification of productive binding modes (near-attack conformations; NACs) for the S_N_2 reaction was based on the distances and angles between the nucleophile and the substrate atoms according to Hur *et al.* (2003 ▸): the distance between one of the nucleophile carboxyl O atoms (Asp95 OD atom; residue numbering in DmmarA) and the halogen-bound C atom has to be *d*_O—C_ ≤ 3.41 Å, with an angle formed by the O, C and halide atoms α_O—C—Br_ ≥ 157°. As reported previously (Daniel *et al.*, 2015 ▸), we also require at least weak hydrogen bonding between the reactive Cl atom and the halide-stabilizing residues, defined by a distance between the halide and the indole polar H atom of Trp96 and the side-chain NH hydrogen of Asn28 *d*_Br—H_ ≤ 3.0 Å. In several cases only quasi-NAC modes were found, where some of the previous geometric requirements were exceeded.

### 2.9. Adiabatic mapping

We studied the potential energy surface along the reaction coordinate using a quantum-mechanics/molecular-mechanics (QM/MM) hybrid approach (Ranaghan & Mulholland, 2010 ▸; Lonsdale *et al.*, 2012 ▸) to evaluate the energetic profiles of the S_N_2 reaction of the two enantiomers of 2-bromopentane with DmmarA and calculate the respective energy barriers, Δ*G* ^‡^. For this, the productive docking binding modes (NACs), identified as described above, were subjected to QM/MM calculations (Walker *et al.*, 2008 ▸) as implemented in *AMBER*. The topology of each structure was prepared by the *tLEaP* module of *AmberTools* 14, using the ff14SB (Maier *et al.*, 2015 ▸) force field for the proteins and the PREPI parameters for the ligand, obtained from the MOL2 files containing the partial atomic charges obtained as described above. The complexes were minimized in vacuum (igb=6). Five rounds of optimization, each consisting of 500 cycles of steepest descent followed by 500 conjugate-gradient cycles, were performed as (i) one step with all heavy atoms restrained with a harmonic force constant of 500 kcal mol^−1^ Å^−2^ and (ii) four steps with decreasing restraints on the protein backbone atoms with force constants of 500, 125, 25 and 1 kcal mol^−1^ Å^−2^. Adiabatic mapping along the reaction coordinate was performed by the *sander* module of *AMBER* 16 (Case *et al.*, 2016 ▸). The QM part of the system contained the ligand molecule, the side chains of the catalytic aspartate (Asp95) and the halide-stabilizing residues (Asn28 and Trp96) and had charge −1. The semi-empirical PM6 Hamiltonian (Stewart, 2007 ▸) was used to treat the QM part of the system and the ff14SB force field was used to treat the MM part. The QM/MM boundary was treated through explicit link atoms and the cutoff for the QM/MM charge interactions was set to 999 Å. The backbone was constrained with a force constant of 1.0 kcal mol^−1^ Å^−2^. The reaction coordinate was defined as the distance between the nearest OD atom of Asp95 and the C atom of the ligand under attack. The reaction coordinate was tracked in decrements of 0.025 Å, each involving 1000 minimization steps of the limited-memory Broyden–Fletcher–Goldfarb–Shanno quasi-Newton algorithm (Zhu *et al.*, 1997 ▸). The total potential energy of the system was extracted from the *AMBER* output files for each step. The energy barrier, Δ*G* ^‡^, was calculated as the difference between the lowest energy of the ground state and the energy of the transition state.

## 3. Results

### 3.1. Quality control suggests a quaternary structure

**Figure 1:**
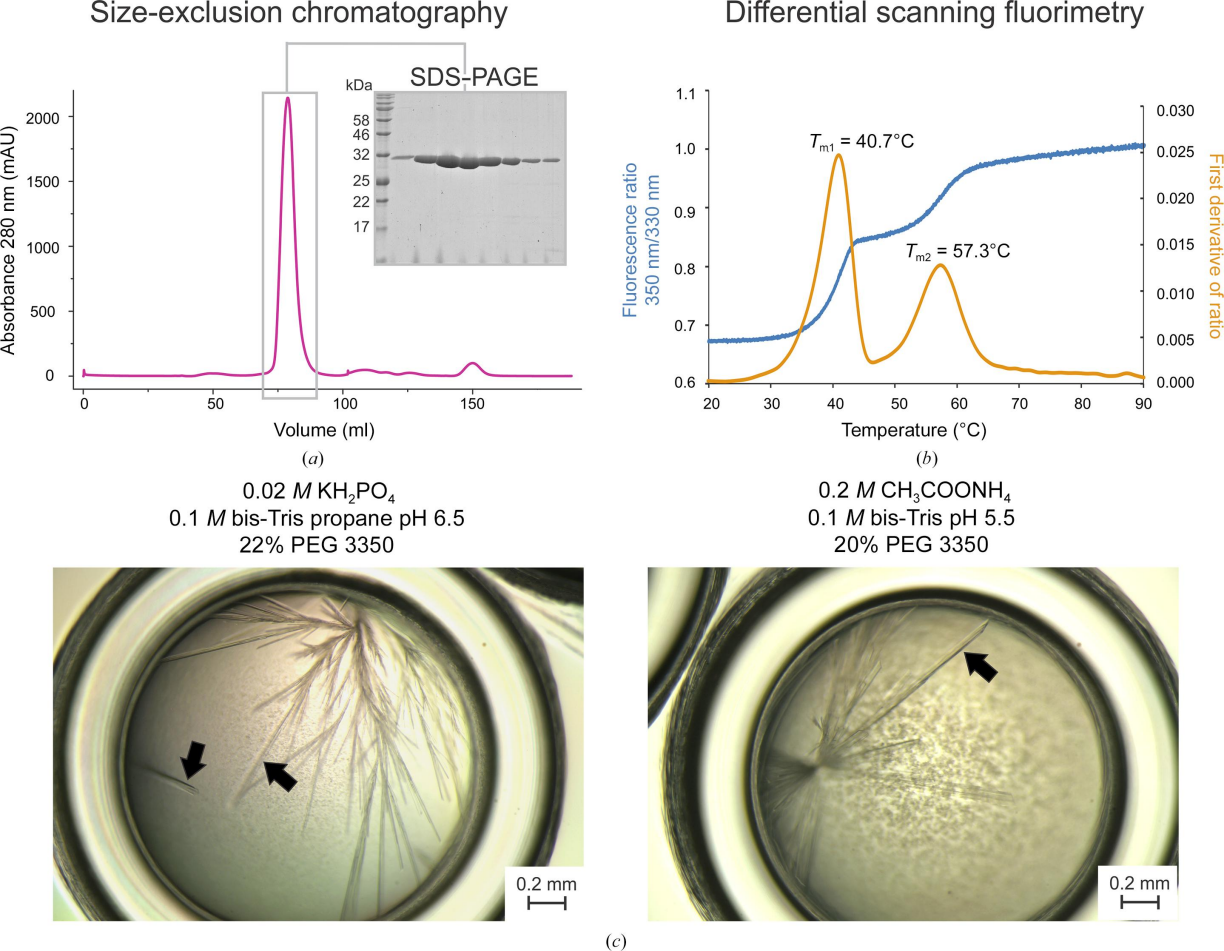
Biochemical characterization and crystal morphology. (*a*) Elution profile from size-exclusion chromatography and SDS–PAGE. (*b*) Thermal unfolding experiments. The blue curve represents the tryptophan fluorescence ratio at 350 nm/330 nm and the orange curve is the first derivative of the ratio. Note that two major melting points accompany the melting of DmmarA: *T*_m1_ = 40.7°C and *T*_m2_ = 57.3°C. (*c*) Micrographs of the needle-like DmmarA crystals obtained at pH 6.5 (left) and pH 5.5 (right). Black arrowheads indicate diffraction-quality crystals that display three-dimensional morphology. Bars represent 0.2 mm.

Recombinantly produced DmmarA enzyme was isolated by a purification procedure that combined metal-affinity and size-exclusion chromatography (SEC). The SEC elution profile of the DmmarA enzyme is shown in Fig. 1 ▸(*a*). The protein was purified to high homogeneity and crystallization quality, as confirmed by SDS–PAGE analysis (Fig. 1 ▸ *a*).

The thermal denaturation profile of DmmarA was measured by nanoDSF. As shown in Fig. 1 ▸(*b*), nanoDSF experiments identified a two-step melting transition with the first transition midpoint at ∼40.7°C, followed by the second transition midpoint at ∼57.3°C. We deduced that the first transition could correspond to dissociation of the DmmarA homodimer, while the second transition could represent the unfolding of separated monomers.

### 3.2. Crystal morphology and diffraction quality

Crystallization screening and subsequent crystal-growth optimization identified two chemical conditions, at pH 6.5 and pH 5.5, where we could reproducibly obtain DmmarA crystals. Unfortunately, all DmmarA crystals that grew were thin and fragile with a needle-like morphology (Fig. 1 ▸ *c*); during the cooling process and handling, most crystals disintegrated into small crystalline needles.

Despite this, the DmmarA crystals showed diffraction quality, complete crystallographic data sets were collected and two independent structures were determined by molecular replacement using the structure of the HLD LinB (PDB entry 4wdq) as a search model. Several cycles of manual building and automatic refinement further refined the initial models. The final models have good deviations from ideal geometry (Table 2 ▸).

The first structure originated from a crystal grown in mother liquor composed of 0.02 *M* KH_2_PO_4_, 0.1 *M* bis-Tris propane pH 6.5, 22% PEG 3350. This crystal structure was solved to a resolution of 1.85 Å in space group *P*12_1_1, with unit-cell parameters *a* = 91.816, *b* = 61.381, *c* = 106.689 Å, α = 90, β = 106.256, γ = 90°. The *Matthews Probability Calculator* (Weichenberger & Rupp, 2014 ▸) suggested four DmmarA (∼33 000 Da) molecules in the asymmetric unit and these were identified in molecular-replacement searches.

The second type of diffracting crystals were obtained in mother liquor composed of 0.2 *M* ammonium acetate, 0.1 *M* bis-Tris pH 5.5, 20% PEG 3350. These crystals diffracted to 1.6 Å resolution and belonged to the same space group *P*12_1_1. The unit-cell parameters were *a* = 90.699, *b* = 60.766, *c* = 104.777 Å, α = 90, β = 105.489, γ = 90°. Similarly, the asymmetric unit contains four DmmarA molecules. Although the resolution of the DmmarA structure from crystals grown at pH 5.5 is better than that obtained at pH 6.5, the electron-density map is poorly resolved in several sites, and for this reason some residues could not be built into density.

### 3.3. The structure of DmmarA shows the canonical HLD fold

**Figure 2:**
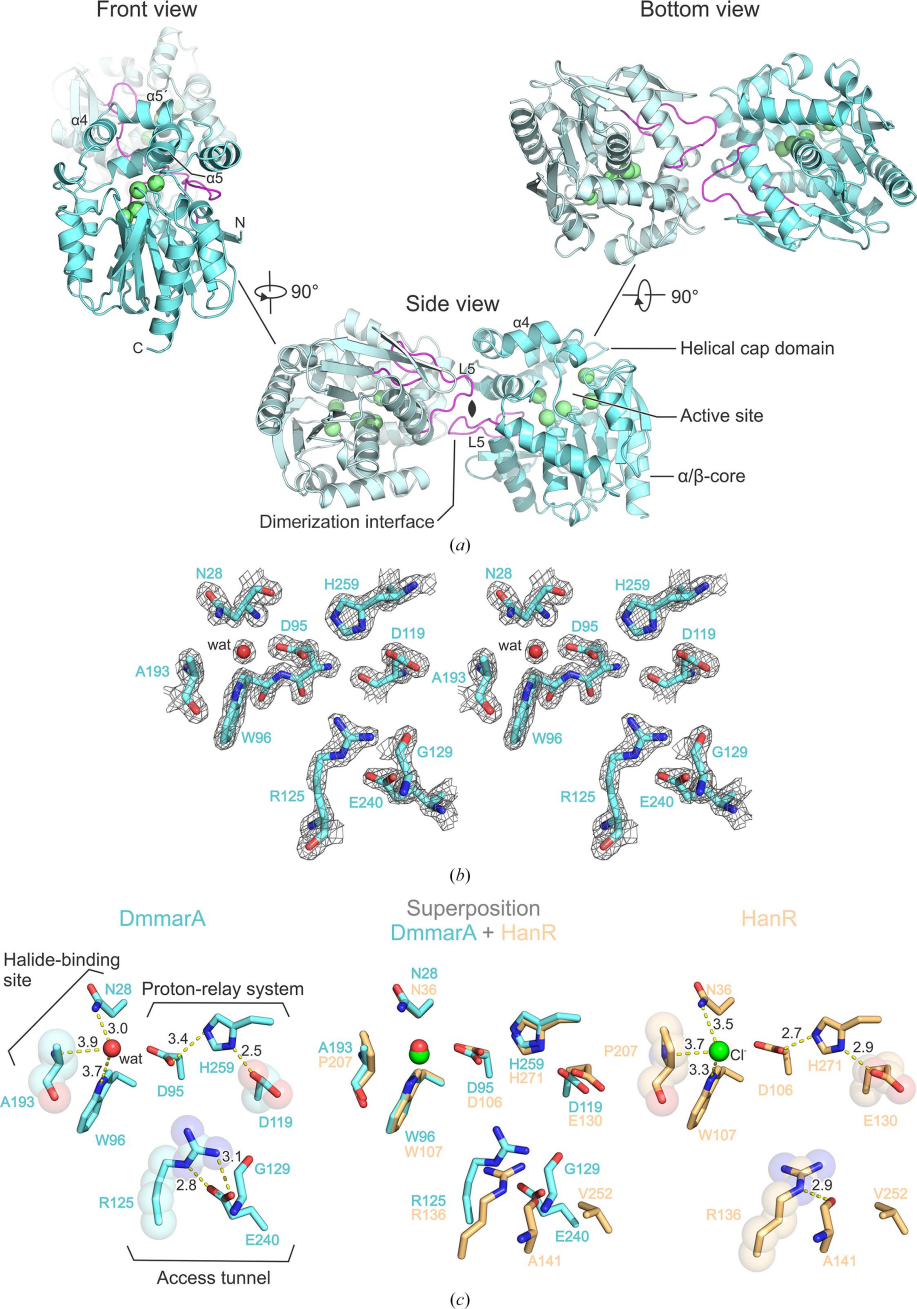
Crystal structure of DmmarA. (*a*) Overall structure of DmmarA. The front view is shown on the left, the side view in the middle and the bottom view on the right. Chain *A* is displayed as a blue cartoon and chain *B* as a light blue cartoon. Amino acids forming the catalytic pentad are visualized as green spheres. The L5 loop is coloured purple. (*b*) Stereo 2*F*_o_ − *F*_c_ electron-density map contoured at 2σ for key active-site residues (grey mesh). Protein residues are shown as blue sticks and water as a red sphere. (*c*) Active sites of DmmarA and HanR. The left panel depicts the active site of DmmarA. Residues in the proton-relay system, halide-binding site and access tunnel are displayed as blue sticks. Unique residues are highlighted as semi-transparent spheres. Hydrogen bonds between residues and to water are shown as yellow dashed lines. Water is visualized as a red sphere. The middle panel displays a superposition of the active sites of DmmarA (blue) and HanR (gold). The right panel shows the active site of HanR. The residues are displayed as gold sticks; hydrogen bonds between residues and the coordination of a chloride ion are shown as yellow dashed lines. The chloride ion is visualized as a green sphere.

**Figure 3:**
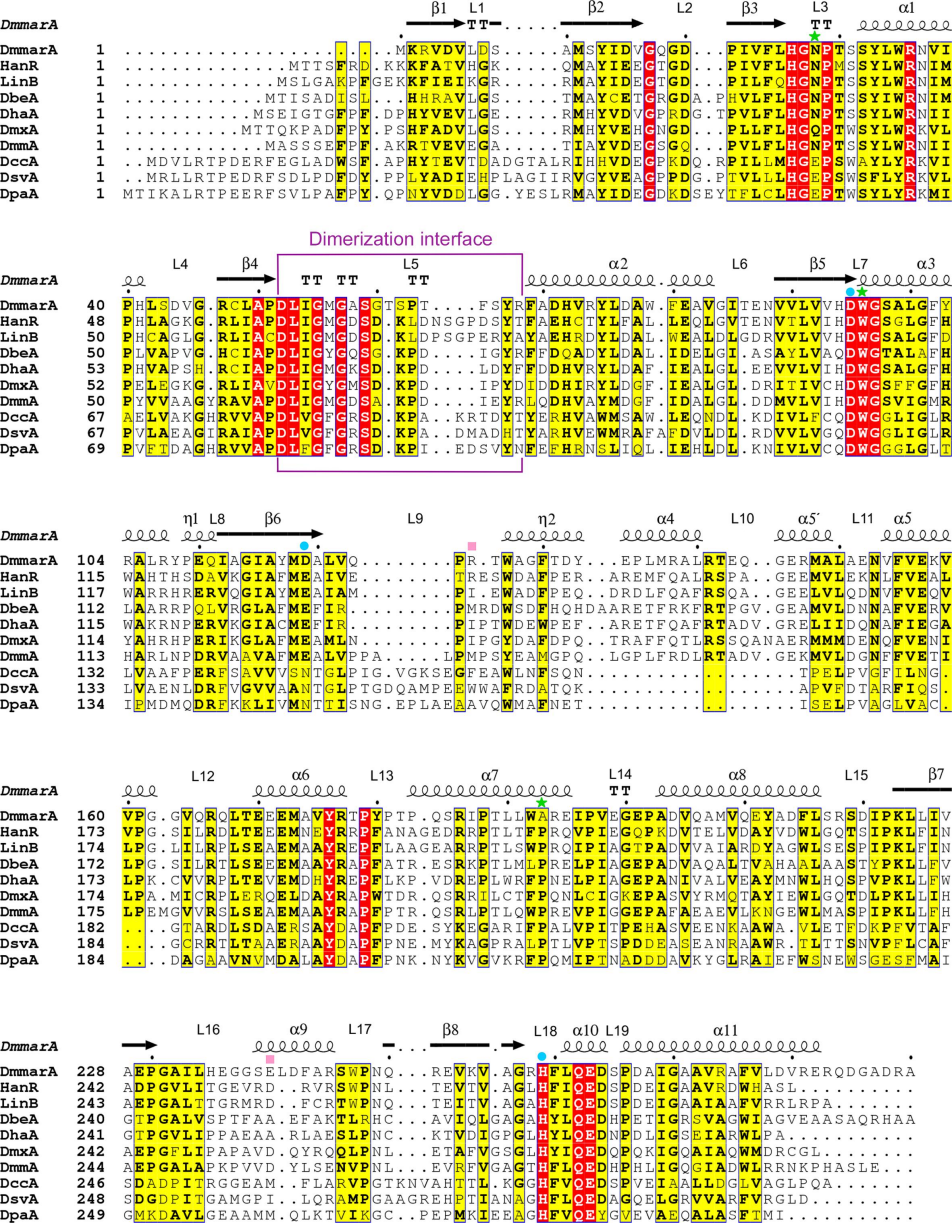
Multiple sequence alignment of different HLDs. The alignment includes the sequences of DmmarA, HanR, LinB, DbeA, DhaA, DmxA, DmmA, DccA, DsvA and DpaA, with the secondary-structure topology of DmmarA shown above the aligned sequences. Residues of the catalytic triad are marked with blue dots, halide-stabilizing residues with green stars and atypical residues present in the catalytic cavity with pink squares. The dimerization interface is framed in purple.

The asymmetric units of both crystal forms of DmmarA (pH 6.5 and pH 5.5) contain four protein chains (*A*–*D*), of which chains *A* and *B* form the first biological homodimer and chains *C* and *D* form the second biological dimer. The overall structure of DmmarA shows a canonical HLD fold that shares major structural features with other HLDs. It consists of a main domain with a typical α/β-hydrolase fold and a helical cap domain (Fig. 2 ▸ *a*). The main domain includes an eight-stranded β-sheet, with the β2 strand being in an antiparallel orientation to the others, surrounded by three helices (α1, α10 and α11) on one side and four helices (α2, α3, α8 and α9) on the other side. The cap domain, which contains six helical regions (η1, α4, α5, α5′, α6 and α7), is anchored by the L9 loop and the L14 loop in between the β6 strand and the α8 helix. Compared with most HLDs, the DmmarA sequence is shortened at its N-terminal end. Therefore, its tertiary structure immediately starts with the β1 strand, lacking the preceding helix frequently found in other HLD family members (Fig. 3 ▸). Pairwise structural comparisons using the *DALI* server (Holm, 2020 ▸) showed that the DmmarA structure is closest to that of the dehalogenase LinB (PDB entry 1mj5; Oakley *et al.*, 2004 ▸), with 54% sequence identity and a root-mean-square deviation (r.m.s.d.) on C^α^ atoms of 0.9 Å (Supplementary Fig. S1).

### 3.4. Zooming in on molecular specificities of the active-site pocket

The active site of DmmarA is located between the main α/β-core and the helical cap domain. In the active site we find the proton-relay catalytic triad, which is atypically composed of a nucleophilic aspartate (Asp95), a base histidine (His259) and a catalytic acid (Asp119) (Figs. 2 ▸ *b* and 2 ▸ *c*). This constellation of the catalytic triad (Asp–His–Asp) is unique among HLD-II subfamily members, as the other enzymes of this subfamily contain glutamate as the catalytic acid (Chovancová *et al.*, 2007 ▸; Fig. 3 ▸). Two canonical halide-stabilizing residues, Asn28 and Trp96, are present in DmmarA. The side chains of Asn28 (∼3.05 Å) and Trp96 (∼3.73 Å) make hydrogen bonds to a water molecule that occupies a site where a halogen ion is typically bound during the dehalogenation reaction.

Surprisingly, DmmarA contains an alanine (Ala193) next to the halide-stabilizing Trp96 instead of the canonical proline, which was always conserved in all HLD enzymes characterized to date (Fig. 3 ▸). This amino-acid change causes a slight shift in the protein backbone, positioning the carbonyl group of Trp192 in proximity to the indole amine N atom of Trp96 (∼3.06 Å). Due to these structural shifts, the bonding distance between the carbonyl O atom of Trp192 and the indole amine of Trp96 (∼3.06 Å) is shorter than the hydrogen bond between the indole amine of the halide-stabilizing Trp96 and the water molecule bound in the halide-binding site (∼3.73 Å).

Another unique residue in the active-site pocket is Arg125. The presence of arginine in this position has previously been observed in the haloalkane dehalogenase HanR (PDB entry 4brz; Novak *et al.*, 2014 ▸). However, the positioning of Arg125 in DmmarA is rather different and this is because of bidentate hydrogen bonding to the side-chain carboxylate of Glu240. The absence of this glutamate in HanR allows the arginine to interact with neighbouring residues in a dissimilar way, significantly changing its spatial positioning. Due to these specificities, the side chain of Arg125 in the structure of DmmarA protrudes deeply towards the active-site pocket, approaching the catalytic centre (∼7.1 Å), while the corresponding arginine in HanR (Arg136) does not. In HanR, the side chain of Arg136 is differently constrained since it makes a hydrogen bond to the backbone carbonyl of Ala141 (Fig. 2 ▸ *c*).

### 3.5. Computational insights on the origin of enantioselectivity

DmmarA has previously revealed enantioselectivity for the (*S*)-enantiomer over the (*R*)-enantiomer of the substrate 2-bromopentane (*E*-value of 6.33; Vasina *et al.*, 2022 ▸). To understand this unusual enantioselectivity, we performed molecular docking with the two isomers of the substrates 2-bromopentane (2-BP) and 2-bromohexane (2-BH). For comparison, we docked the same compounds into the (*R*)-enantioselective enzyme DbjA, which has one of highest *R*-enantioselectivities among the HLD family (*E*-value of 132 with 2-BP; Prokop *et al.*, 2010 ▸; Liskova *et al.*, 2017 ▸). The differences in the binding energies for the *R* and *S* isomers were not very significant (Supplementary Table S1). Nonetheless, although small, the theoretical ratios between their thermodynamic stability consistently followed the order of the known preferences for the two tested enzymes (*R*/*S* ratio of <1.0 for DmmarA and >1.0 for DbjA; Supplementary Table S1).

**Figure 4:**
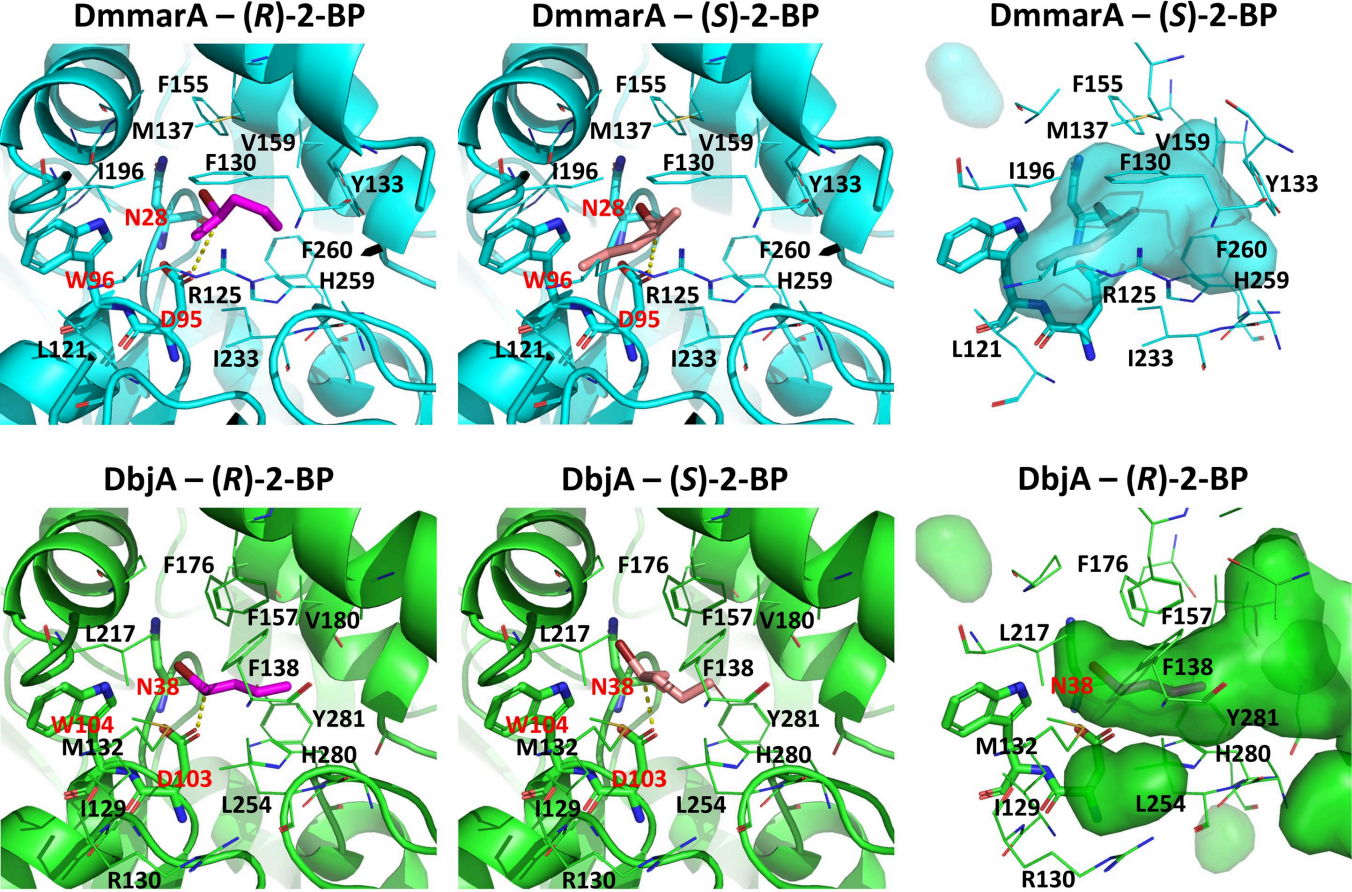
Productive binding modes obtained by molecular docking. The results for DmmarA (top, cyan) and DbjA (bottom, green) with (*R*)-2-BP (left, magenta sticks) and (*S*)-2-BP (centre, salmon sticks) are represented; the active-site pockets of the enzymes are shown with the respective preferred enantiomers of 2-BP (right). The residues neighbouring the ligands are labelled. The residues involved in the S_N_2 reaction (sticks) are labelled in red: the nucleophile (Asp103 or Asp95) and the halide-stabilizing residues (Asn28 and Trp96 for DmmarA or Asn38 and Trp104 for DbjA). The distance between the carboxylic O atom and C atom involved in the S_N_2 reaction is shown as a dotted yellow line. All figures are presented from the same vantage point.

We analysed the productive binding modes (Fig. 4 ▸ and Supplementary Fig. S2) by calculating near-attack conformations (NACs). The results were very similar for 2-BP and 2-BH, depending mostly on the enzyme and the enantiomer. In some cases only quasi-NAC binding modes were found, which may hint at some difficulties in achieving good reactivity with these substrates, for example for DmmarA with (*R*)-2-BP and (*R*)-2-BH (Supplementary Table S1). The orientations of the *R* and *S* enantiomers were not significantly different in DbjA, whereas in DmmarA the two isomers adopted very different binding modes in the active site (Fig. 4 ▸). This seems to be due to a larger subpocket found in DmmarA around Ile196 and Leu121, which promotes binding of the *S* substrates in this region. This subpocket is not available in DbjA due to the presence of the bulkier Leu217, Ile129 and Met132 instead of Ile196, Leu121 and Arg125, respectively (Fig. 4 ▸). Such differences in the active sites may explain their different propensities to bind and ultimately to catalyse reaction of the (*R*)- and (*S*)-enantiomers.

To predict more accurately how the first chemical step (the S_N_2 reaction) in DmmarA could differ for (*R*)- and (*S*)-2-BP, we performed QM/MM adiabatic mapping along the reaction coordinate, as reported previously for other haloalkane dehalogenases (Marques *et al.*, 2022 ▸). We could estimate the respective activation energies as Δ*G* ^‡^ = 14.7 kcal mol^−1^ for (*R*)-2-BP and 9.9 kcal mol^−1^ for (*S*)-2-BP. The activation barrier was higher for the (*R*)-enantiomer than for the (*S*)-enantiomer by 4.8 kcal mol^−1^, which can be translated into a 3.3 × 10^3^-fold faster reaction rate for the *S* substrate. This preference is qualitatively in agreement with the experimental findings for 2-BP (Vasina *et al.*, 2022 ▸) and confirms that the active site of DmmarA is more suitable to catalyse reaction with the (*S*)-enantiomer.

### 3.6. Unusual mode of homodimerization through the L5 loop

**Figure 5:**
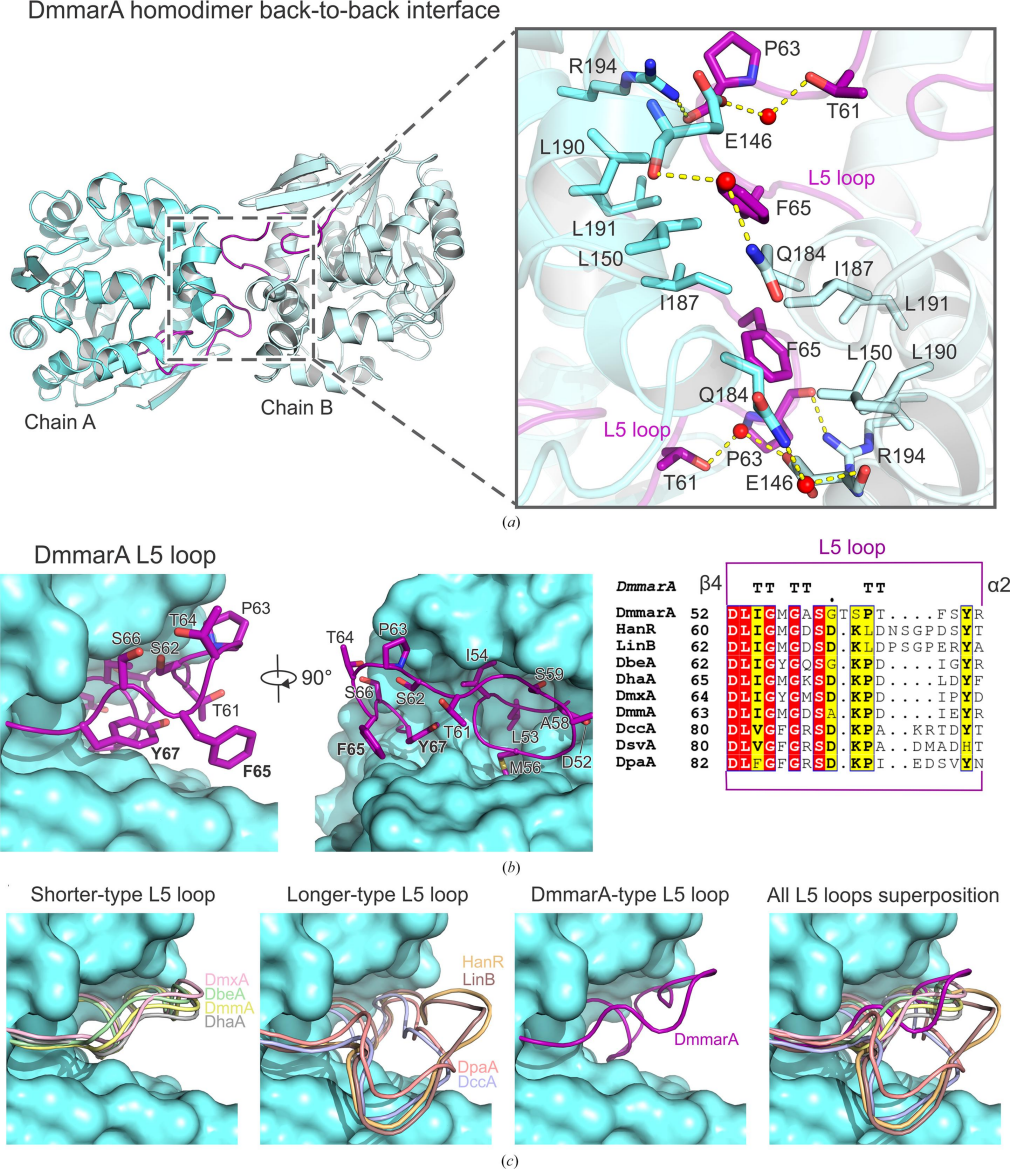
Dimerization through the L5 loop. (*a*) Back-to-back dimerization interface of DmmarA. Chain *A* is displayed as a blue cartoon and chain *B* as a light blue cartoon. The L5 loop is coloured purple. In the close-up view, interacting residues are shown as sticks. Waters are visualized as red spheres and bonds as yellow dashed lines. (*b*) The unique DmmarA L5 loop. The loop is shown in purple and the residues of the loop are shown as purple sticks. The rest of the enzyme is visualized as a blue surface. The right panel shows a multiple sequence alignment of the L5 loop in different HLDs. (*c*) Types of L5 loop conformation. On the left, the shorter-type L5 loop of DbeA (green), DhaA (grey), DmxA (pink) and DmmA (yellow) is depicted. The second panel shows the longer-type L5 loop of HanR (gold), LinB (brown), DccA (blue-purple) and DpaA (salmon). The third panel shows the DmmarA-type L5 loop (purple). A superposition of all L5 loops is displayed in the right panel.

Careful inspection of the electron-density maps revealed an atypical mode of DmmarA homodimerization that has not previously been observed. Precisely, the DmmarA structure displays a new so-called back-to-back homodimerization mode (Fig. 5 ▸ *a*), distinguishing it from the previously described HLD dimers. The DmmarA homodimer is assembled due to noncovalent interactions between the L5 loop located in the main α/β-core, the α5′ and α7 helices in the cap domain of one chain and the corresponding symmetric interface of the second chain. A comprehensive analysis of interfaces between interacting chains using the *PLIP* tool (Adasme *et al.*, 2021 ▸) revealed two hydrogen bonds, eight hydrophobic interactions and two water bridges. Hydrogen bonding occurs between the main-chain carbonyl of Pro63 (L5 loop, chain *A*) and the side-chain N atom of Arg194 (α7, chain *B*), with a distance of 2.75 Å. The same bond exists due to the near-perfectly symmetrical nature of the dimerization interface between the carbonyl of Pro63 (L5, chain *B*) and the side chain of Arg194 (α7, chain *A*), with a distance of 2.8 Å. Furthermore, multiple hydrophobic and nonpolar interactions maintain the dimeric assembly. Notably, the side chain of Phe65 in the L5 loop (chain *A*) plays a dominant role here, as it makes contacts with Leu150 (3.91 Å), Ile187 (3.35 Å), Leu190 (4.04 Å) and Leu191 (3.96 Å) from chain *B*. Analogous hydrophobic interactions are observed on the opposite side of the twofold rotational axis. Additionally, several water-mediated bridges are formed, for example between the main-chain carbonyl of Glu146 (α5′, chain *A*) and the amide N atom of Gln184 (α7, chain *B*). Another water bridge is seen between the side-chain hydroxyl of Thr61 (L5, chain *B*) and the side-chain carboxylate of Glu146 (α5′, chain *A*), with distances of 2.65 and 2.54 Å, respectively.

Computational mapping of the dimerization interface using *PISA* interface calculations (Krissinel & Henrick, 2007 ▸) revealed a relatively small interface solvent-accessible area (618 Å^2^) compared with the total solvent-accessible area (10 907.7 Å^2^) and a complex-formation significance score (CSS) of 0, suggesting no biologically relevant oligomeric assembly. In contrast, all of the other HLD dimers determined to date showed a CSS above 0, usually larger interfaces and oligomeric assemblies (Table 3 ▸). *PISA* calculated a negative free energy of dissociation of the dimer (Δ*G* ^diss^ = −4.6 kcal mol^−1^, with a concomitant *K*_d_ = 2.3 × 10^3^), which indicates that the associated dimer is predicted to be thermodynamically unstable. Thus, *PISA* and CSS did not support a dimeric form of DmmarA. Moreover, the DmmarA structure is most closely structurally related to that of the dehalogenase LinB, which is a well characterized monomeric enzyme. In contrast, however, predicting the DmmarA structure using *AlphaFold*2 (Mirdita *et al.*, 2022 ▸) resulted in a very similar dimeric assembly and active-site residue conformation to our crystallographic structure (Supplementary Fig. S3).

### 3.7. A SAXS experiment confirms homodimeric association in a solution

**Figure 6:**
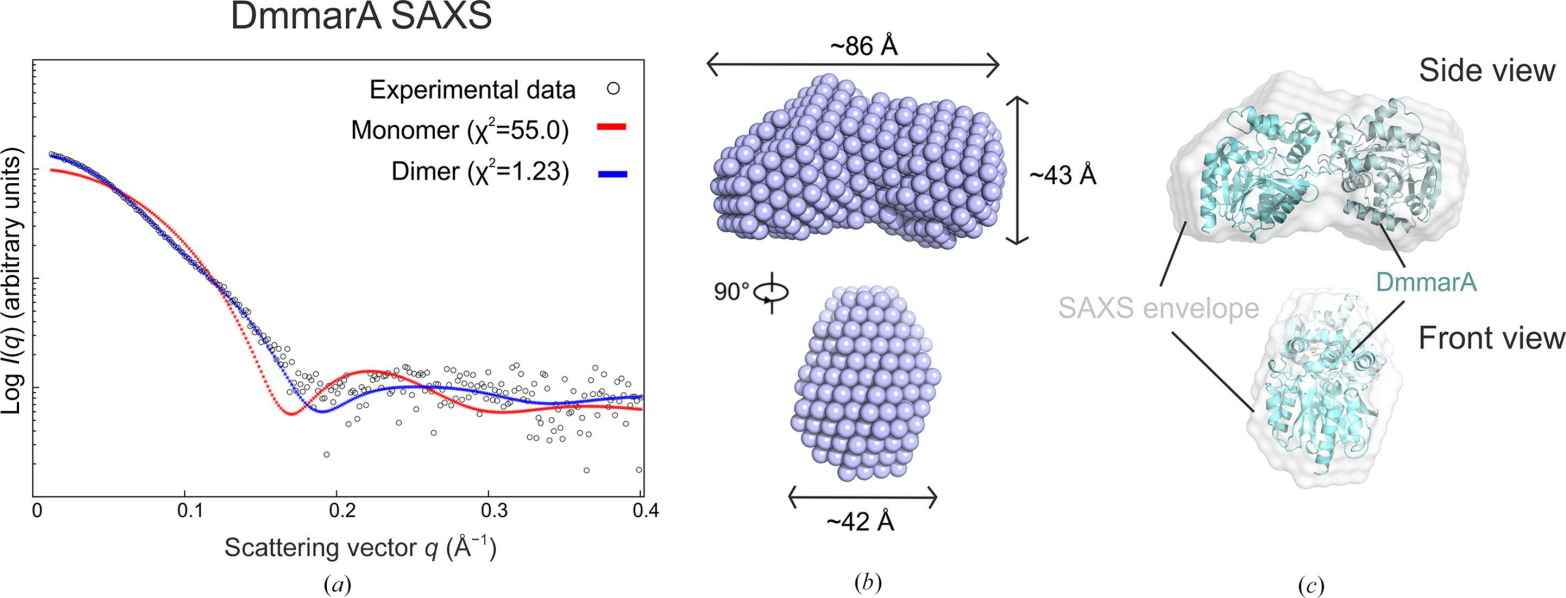
SAXS solution structure of DmmarA. (*a*) The experimental SAXS scattering curve (dots) is shown with the calculated curves for the monomer (red line) and dimer (blue line). (*b*) *Ab initio* model of DmmarA (purple spheres). (*c*) The *ab initio* envelope (semi-transparent spheres) is superposed with the DmmarA crystal dimer (blue cartoon).

Next, we probed the SEC-purified DmmarA enzyme using the small-angle X-ray scattering (SAXS) technique to determine its structure in solution. The SAXS experimental data perfectly fit the calculated scattering curve of the dimeric DmmarA crystal model with χ^2^ = 1.2. Moreover, the *ab initio* model envelope perfectly matches the dimeric structure of DmmarA obtained by X-ray crystallography (Fig. 6 ▸). In contrast, the experimental scattering profile does not correspond to the calculated curve of the monomeric DmmarA structure (χ^2^ = 55). Taken together, the SAXS analysis provided direct experimental evidence of a dimeric nature of DmmarA.

### 3.8. The expanding dimerization modes used in the HLD family

**Figure 7:**
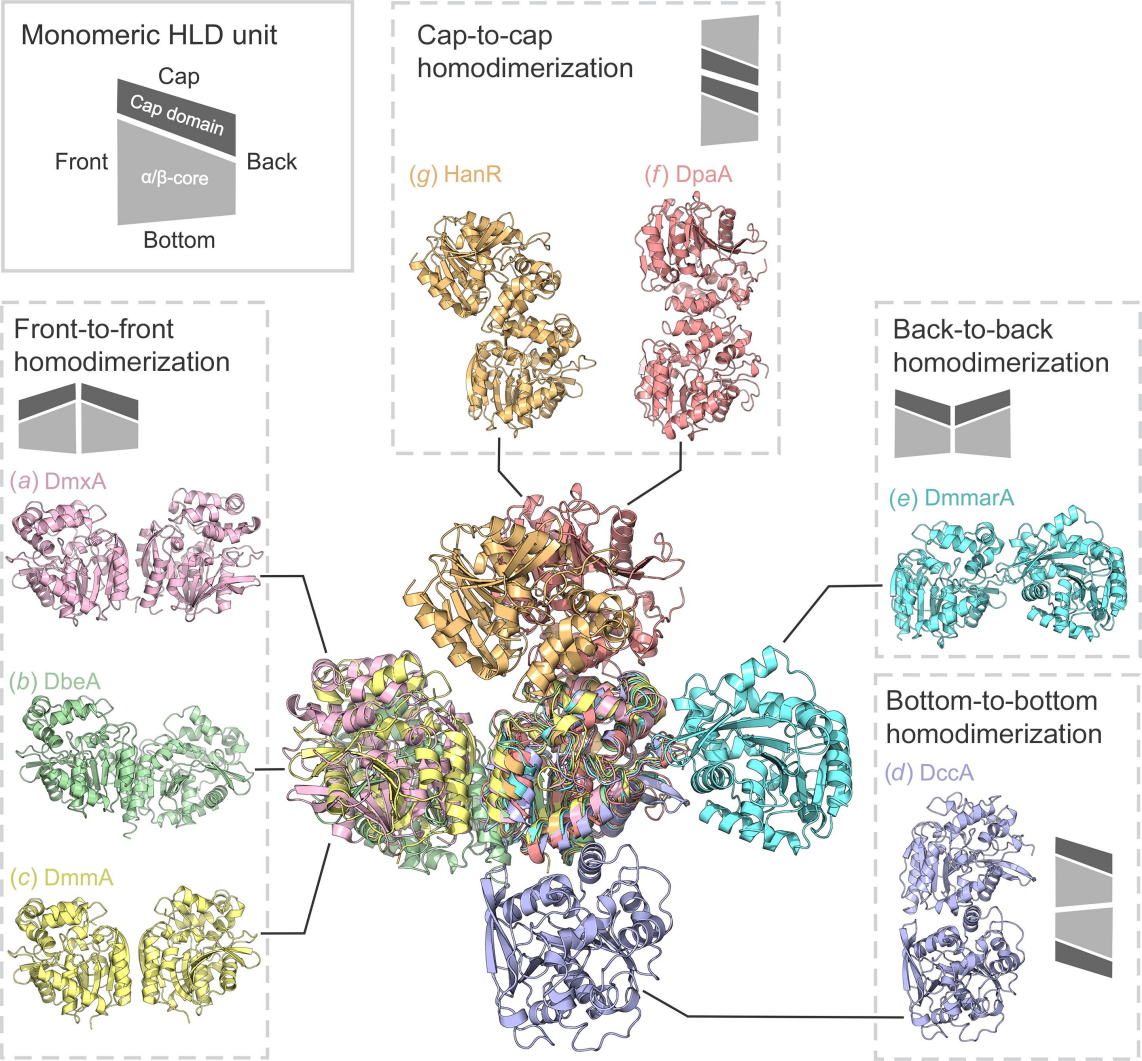
Diverse dimerization modes in the HLD family. The superposition of HLD dimers is depicted in the centre. Front-to-front interactions of (*a*) DmxA (pink), (*b*) DbeA (green) and (*c*) DmmA (yellow) are displayed on the right, the bottom-to-bottom interaction of (*d*) DccA (blue-purple) is displayed at the bottom, the back-to-back dimerization interface of (*e*) DmmarA (blue) is shown on the left and the cap-to-cap interaction of (*f*) DpaA (salmon) and (*g*) HanR (gold) is shown at the top. The grey schemes illustrate the different dimerization modes, with the darker grey polygons representing cap domains and the lighter grey polygons representing main domains.

The well described HLDs form dimers using three different protein–protein interfaces that can be termed (i) front-to-front dimerization (DbeA, DmmA, DmxA and DbjA), (ii) bottom-to-bottom dimerization (DccA) and (iii) cap-to-cap dimerization (DpaA, HanR and DatA). A structural comparison of seven experimentally determined HLD dimers is shown in Fig. 7 ▸. The dehalogenase DbeA forms dimers by front-to-front dimerization, in which the C-terminal α-helices play a key role (Chaloupkova *et al.*, 2014 ▸), while the DmmA chains interact through the α10 and β8 secondary structures (Gehret *et al.*, 2012 ▸). Dimerization of DmxA is mediated by the C-terminal helix and β8 sheet, with the help of a disulfide bridge between cysteine residues of each monomer (Chrast *et al.*, 2019 ▸). DccA mainly uses bottom-to-bottom protein interaction of α8 helices in the main domains. The enzymes DpaA and HanR dimerize through cap-to-cap interaction, DpaA employs noncovalent interactions of the α5 and α6 helices (Mazur *et al.*, 2021 ▸), and the HanR dimer is strictly formed by interactions in the helical cap domain. The DmmarA structure determined in this study thus reveals a new, fourth type of self-association interface that leads to so-called back-to-back dimerization, in which the L5 loop plays a dominant role.

### 3.9. The specific composition and conformation of the L5 loops drive back-to-back dimerization

There was a fundamental question as to why the DmmarA enzyme adopted the new back-to-back dimerization interface and not those that have previously been observed in other members of the HLD family­. A careful inspection of superposed structures revealed a specific conformation of the L5 loop in DmmarA that distinguishes it from other HLDs (Fig. 5 ▸ *c*). In general, L5 loops in HLDs can be divided into two different clusters based on sequence and structural comparisons. The first cluster represents enzymes with a shorter L5 loop (18 residues; DbeA, DhaA, DmxA and DmmA), while the second cluster comprises enzymes with a relatively longer L5 loop (20–22 residues; HanR, LinB, DccA and DpaA). DmmarA does not belong to either of those clusters, as its L5 loop shows unique features. As shown in Fig. 5 ▸(*b*), the L5 loop of DmmarA has a specific sequence composition; the presence of a phenylalanine at position 65 in particular is very unusual. Structurally, the bulky aromatic side chain of Phe65 protrudes out of the surface of the protein because the surface subpocket nearby is occupied by the conserved Tyr67. Moreover, the specific conformation of the L5 loop of DmmarA has an additional cause. The overall DmmarA sequence is truncated at its N-terminal end; it starts immediately with the β1 strand, with no N-terminal flanking region. In some HLDs (for example DccA and DpaA), the very N-terminal structural elements are spatially in contact with the L5 loop, affecting its conformational behaviour, which is not the case in the DmmarA structure. Together, we consider that the unique sequence and conformation of the L5 loop in DmmarA confer the emergence of a new type of dimerization interface.

## 4. Discussion

In this study, we determined high-resolution crystallographic structures of the (*S*)-enantioselective haloalkane dehalogenase DmmarA from the vertebrate-pathogenic *M. marinum*. Crystallization screenings always yielded tiny fragile needle-like crystals that were obtained in various chemical conditions. Despite this, two diffraction data sets at 1.6 and 1.85 Å resolution were collected from crystals originating from different mother liquors and X-ray structures were solved by molecular replacement.

The overall structure of DmmarA shows the canonical HLD fold, with the highest similarity to the well characterized dehalogenase LinB (r.m.s.d. on C^α^ atoms of 0.9 Å). However, several structural dissimilarities are visible. Interestingly, the DmmarA enzyme exhibits an (*S*)-enantiopreference (Vasina *et al.*, 2022 ▸), which distinguishes it from other members of the HLD family that usually possess an (*R*)-enantiopreference (Vasina *et al.*, 2022 ▸). Our X-ray structures provided unprecedented molecular views of the DmmarA catalytic apparatus that include (i) an atypical composition of the proton-relay catalytic triad (Asp–His–Asp), (ii) the presence of alanine at position 193, where a proline residue is canonically located immediately next to the halide-stabilizing tryptophan, and (iii) a unique constellation of residues lining the active-site pocket. Specifically, the presence and positioning of Arg125 in the active-site pocket is unusual, and together with the atypical composition of the catalytic triad can drive nontraditional (*S*)-enantioselectivity for this enzyme family. Apart from DmmarA, an arginine in this position is only observed in the haloalkane dehalogenase HanR (PDB entry 4brz), and it has been suggested that it can stabilize the distal halogen group of long dihalogenated compounds (Novak *et al.*, 2014 ▸). Moreover, molecular docking and QM/MM calculations on the S_N_2 reaction suggest that the active site of DmmarA is more suitable to bind and catalyse the dehalogenase reaction for the (*S*)-enantiomer of 2-bromopentane compared with the (*R*)-enantiomer.

Another structural feature that distinguishes DmmarA from other HLDs is its truncated N-terminal end that starts immediately with the β1 strand, with no flanking N-terminal parts. The absence of flanking N-terminal parts has a distant consequence, allowing the L5 loop to adopt a unique conformation in DmmarA. Our crystallographic analysis, supported by nanoDSF and SAXS experiments, provides evidence that the DmmarA enzyme forms symmetric homodimers, predominantly due to the specific composition and conformation of the L5 loop. The self-association mode of DmmarA, termed back-to-back dimerization, is unique among all known dimeric HLDs characterized to date. The DmmarA structures determined in this study thus expand our knowledge of the dimerization potential of HLD-fold proteins. The previously observed self-association modes include front-to-front dimerization (DbeA, DmmA, DmxA and DbjA), bottom-to-bottom dimerization (DccA) and cap-to-cap dimerization (DpaA, HanR and DatA). The self-association of DmmarA through back-to-back dimerization is mediated by interactions between the L5 loop in the main domain and the α5′ and α7 helices in the cap domain of one monomer with the symmetric interface of the second monomer. The utilization of these secondary structures for the formation of an oligomer has not been described in other dimeric structures of HLDs. Sequence and structural comparisons of L5 loops amongst HLDs dimers revealed a different conformation of this loop in DmmarA because of the presence of the bulky aromatic side chain of Phe65 in a position where other HLDs have small residues, for example, leucine or isoleucine. The bulky phenylalanine in DmmarA (Phe65) is unusually exposed on the protein surface. It is therefore attracted to interact with another enzyme molecule to shield its solvent-exposed hydrophobic/aromatic moiety in the symmetric homodimer interface.

## 5. Conclusions

The novel haloalkane dehalogenase DmmarA from the waterborne pathogenic microbe *M. marinum* was successfully crystallized and high-resolution X-ray structures were determined. The structures of DmmarA revealed features that distinguishes it from other members of the HLD family. The most striking molecular features are (i) an atypical architecture of the enzymatic pocket that displays an unusual (*S*)-enantiopreference and (ii) a new mode of so-called back-to-back dimerization due to the unique composition and conformation of the L5 loop. Our findings thus highlight key molecular features that distinguish the DmmarA enzyme from other HLD-family members and provide the structural basis for the design of inhibitors to impair dehalogenation pathways in this vertebrate-infecting microbe.

## 6. List of abbreviations

CSS, complex-formation significance score; FPLC, fast protein liquid chromatography; HLD, haloalkane dehalogenase; IPTG, isopropyl β-d-1-thiogalactopyranoside; LB, Luria broth; LCP, lipidic cubic phase; MM, molecular mechanics; NAC, near-attack conformation; nanoDSF, nano differential scanning fluorimetry; OD, optical density; PDB, Protein Data Bank; PEG, polyethylene glycol; *PISA*, *Protein Interfaces, Surfaces and Assemblies*; QM, quantum mechanics; r.m.s.d., root-mean-square deviation; RCSB, Research Collaboratory for Structural Bioinformatics; SAXS, small-angle X-ray scattering; SDS–PAGE, sodium dodecyl sulfate–polyacrylamide gel electrophoresis; SEC, size-exclusion chromatography; SLS, Swiss Light Source; UFF, Universal Force Field; *XDS*, *X-ray Detector Software*; 2-BP, 2-bromopentane; 2-BH, 2-bromohexane.

## Supplementary Material

PDB reference: DmmarA at pH 6.5, 8b5k

PDB reference: at pH 5.5, 8b5o

Supplementary Table and Figures. DOI: 10.1107/S2059798323006642/jc5060sup1.pdf

## Figures and Tables

**Table 1:** Oligomeric proteins from the HLD-I and HLD-II subfamilies

Enzyme	Organism	PDB code	Oligomeric form	Reference
DpaA	*Paraglaciecola agarilytica* NO2	7avr	Tetramer	Mazur *et al.* (2021 ▸)
DppA	*Plesiocystis pacifica* SIR-1	2xt0	Dimer	Hesseler *et al.* (2011 ▸)
DatA	*Agrobacterium fabrum* C58	3wi7	Dimer	Hasan *et al.* (2011 ▸)
DbeA	*Bradyrhizobium elkanii* USDA 94	4k2a	Dimer	Chaloupkova *et al.* (2014 ▸)
DmmA	Unidentified marine microbiome	3u1t	Dimer	Gehret *et al.* (2012 ▸)
DmxA	*Marinobacter* sp. ELB17	5mxp	Dimer	Chrast *et al.* (2019 ▸)
DccA	*Caulobacter vibrioides* CB15	5esr	Dimer	Carlucci *et al.* (2016 ▸)
HanR	*Rhodobacteraceae* bacterium UDC319	4brz	Dimer	Novak *et al.* (2014 ▸)
DbjA	*Bradyrhizobium diazoefficiens* USDA 110	3afi	Dimer	Sato *et al.* (2005 ▸)

**Table 2:** Data-collection and refinement statistics Values in parentheses are for the highest resolution shell.

	DmmarA at pH 6.5	DmmarA at pH 5.5
Crystallization conditions	0.02 *M* KH_2_PO_4_, 0.1 *M* bis-Tris propane pH 6.5, 22% PEG 3350	0.2 *M* ammonium acetate, 0.1 *M* bis-Tris pH 5.5, 20% PEG 3350
Buffer composition	50 m*M* sodium formate, 10 m*M* Tris pH 8.5	50 m*M* sodium formate,10 m*M* Tris pH 8.5
Wavelength (Å)	1.0	1.0
Temperature (K)	100	100
Resolution range (Å)	44.07–1.849 (1.916–1.849)	42.25–1.597 (1.654–1.597)
Space group	*P*12_1_1	*P*12_1_1
*a*, *b*, *c* (Å)	91.816, 61.381, 106.689	90.699, 60.766, 104.777
α, β, γ (°)	90, 106.256, 90	90, 105.489, 90
Matthews coefficient (Å^3^ Da^−1^)	2.19	2.11
Total reflections	652916 (58413)	960789 (85869)
Unique reflections	97547 (9612)	144159 (14080)
Multiplicity	6.7 (6.1)	6.7 (6.1)
Completeness (%)	99.77 (98.39)	98.83 (96.90)
Mean *I*/σ(*I*)	10.36 (1.20)	11.39 (1.43)
Wilson *B* factor (Å^2^)	22.24	17.78
*R*_merge_	0.143 (1.441)	0.1048 (1.16)
CC_1/2_	0.997 (0.467)	0.998 (0.519)
Reflections used in refinement	97547 (9578)	144159 (14074)
Reflections used for *R*_free_	4800 (497)	7081 (677)
*R*_work_	0.1910 (0.3318)	0.1977 (0.2860)
*R*_free_	0.2409 (0.3623)	0.2380 (0.3081)
No. of non-H atoms		
Total	9668	9744
Macromolecules	8987	8949
Ligands	102	85
Solvent	579	710
Protein residues	1138	1134
R.m.s.d.s		
Bond lengths (Å)	0.010	0.016
Angles (°)	1.00	1.32
Ramachandran statistics (%)		
Favoured	95.74	95.06
Allowed	4.26	4.31
Outliers	0.00	0.63
Rotamer outliers (%)	0.21	0.32
Clashscore	3.45	6.43
Average *B* factor (Å^2^)		
Overall	28.11	29.28
Macromolecules	27.63	28.92
Ligands	43.26	42.39
Solvent	32.87	32.24
PDB code	8b5k	8b5o

**Table 3:** *PISA* interface calculations for the studied DmmarA, the monomeric DhaA and LinB and the oligomeric DbeA, DccA, DmmA, DmxA, DpaA and HanR

Enzyme	Total solvent-accessible area (Å)^2^	Interface solvent-accessible area (Å^2^)	Interface solvent-accessible area (%)	Complex-formation significance (CSS) score	Role in complex formation	Most probable assembly
DmmarA	10907.7	618.0	5.7	0.0	No	Monomer
DhaA	11714.3	499.6	4.3	0.0	No	Monomer
LinB	11627.2	445.0	3.8	0.0	No	Monomer
DbeA	12136.3	935.7	7.7	0.254	Yes	Dimer
DccA	12308.9	947.2	7.7	0.066	No	Dimer
DmmA	11279.2	710.8	6.3	0.160	Yes	Tetramer
DmxA	11628.5	610.6	5.3	0.106	Yes	Dimer
DpaA	12258.1	952.6	7.8	0.483	Yes	Tetramer
HanR	11272.2	957.1	8.5	0.034	No	Dimer
