# Supplementary material for: Atypical homodimerization revealed by the structure of the (S)-enantioselective haloalkane dehalogenase DmmarA from Mycobacterium marinum
Source: Acta Crystallogr D Struct Biol. 2023 Oct 20;79(Pt 11):956–70. doi: 10.1107/S2059798323006642 (PMC10619424; doi:10.1107/S2059798323006642)
Supplement: Supplementary file 1 [file d-79-00956-sup1.pdf]

STRUCTURAL
BIOLOGY

Volume 79 (2023)

Supporting information for article:

Atypical homodimerization revealed by the structure of (*S*)-enantioselective haloalkane dehalogenase DmmarA from *Mycobacterium marinum*

Karolina Snajdarova, Sérgio M. Marques, Jiri Damborsky, David Bednar and Martin Marek

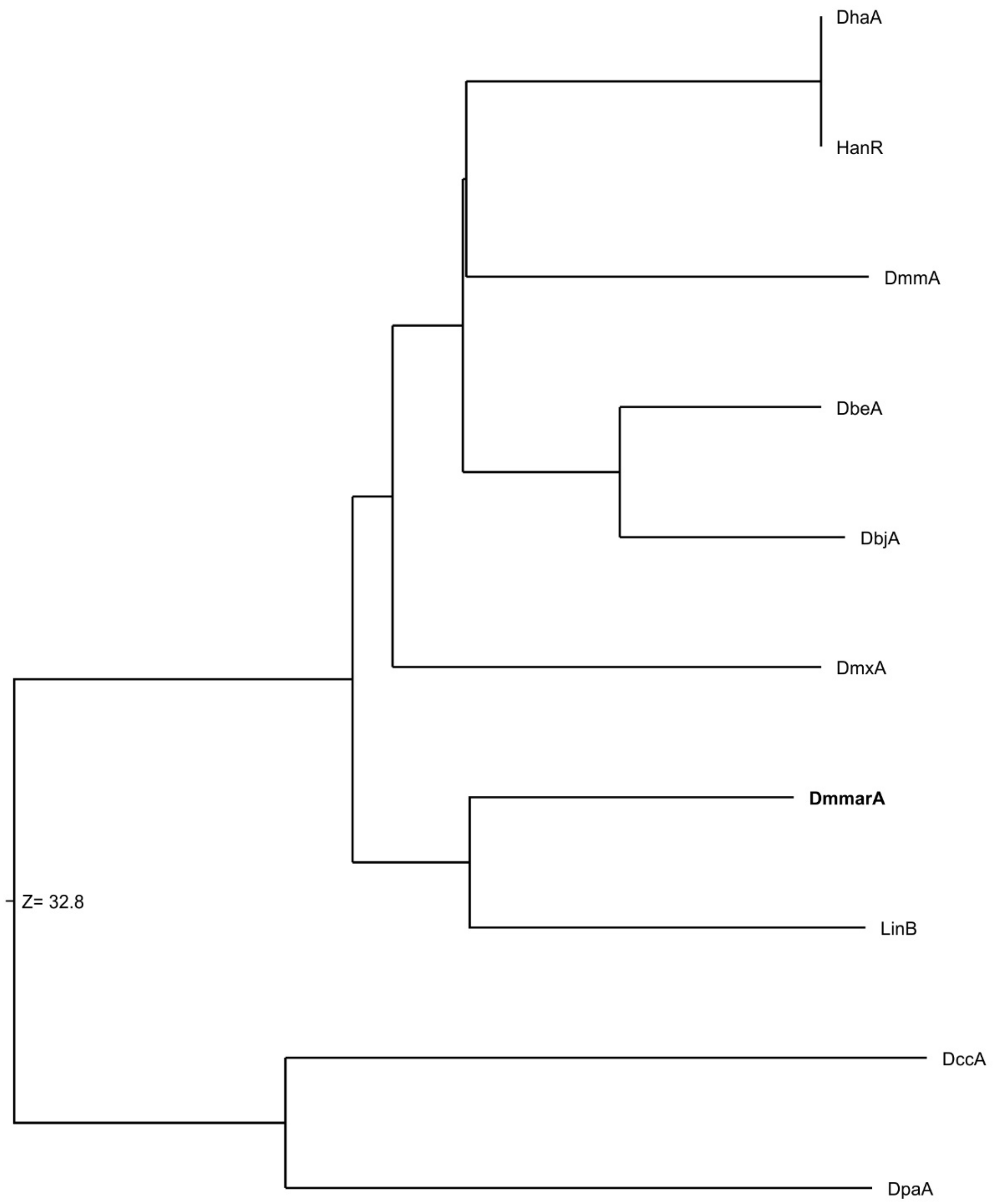

Figure S1 DALI server dendogram of different HLDs.

Table S1 Binding energies (ΔG_{bind}) of the productive binding modes calculated with AutoDock Vina 1.1 and scored with Vina and Smina scoring functions.

		**Vina**		**Smina**		**Kinetic constants**[c]	
**Enzyme**	**Substrate**	**ΔG_{bind}** [a] **(kcal/mol)**	***R/S* ratio**[b]	**ΔG_{bind}** [a] **(kcal/mol)**	***R/S* ratio**[b]	**k_{cat} (s^{-1})**	**K_m (mM)**
DmmarA	(*R*)-2-BP	-3.8*	<0.84	-3.705*	<0.69	0.047 [d]	0.105 [d]
	(*S*)-2-BP	-3.9		-3.923		0.30 [d]	0.0921 [d]
	(*R*)-2-BH	-4.4*	<0.71	-4.299*	<0.60	n.d.	n.d.
	(*S*)-2-BH	-4.6		-4.600		n.d.	n.d.
DbjA	(*R*)-2-BP	-4.0	1.96	-3.944	1.64	0.269 [e]	0.0100 [e]
	(*S*)-2-BP	-3.6		-3.652		0.55 [e]	1.28 [e]
	(*R*)-2-BH	-4.4	1.40	-4.357	1.35	n.d.	n.d.
	(*S*)-2-BH	-4.2		-4.177		n.d.	n.d.

[a]Scores for the best productive docking modes corresponding to the near-attack conformations (*NAC*); *refers to *quasi-NAC*, where some distances or angles were beyond the acceptable values and no better conformations were found.

[b] *R/S ratio* is the stability ratio of the enzyme complexes with (*R*)- over the (*S*)-enantiomer, and is given by $e^{-\frac{\Delta G^R}{RT}}/e^{-\frac{\Delta G^S}{RT}}$, where ΔG^R and ΔG^S are the docking binding energies of the respective best *NAC* modes with the *R* and *S* isomers; RT = 0.593 kcal/mol at 298 K.

[c] Experimental steady-state kinetic parameters; n.d. means “not determined”

[d] Kinetic constants for DmmarA, as reported by (Vasina *et al.*, 2022).

[e] Kinetic constants for DbjA measured by (Liskova *et al.*, 2017).

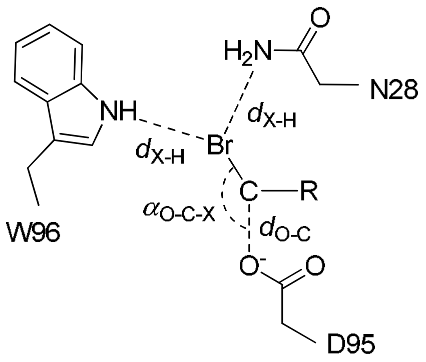

Figure S2 Geometry of the near-attack conformation (*NAC*) of a general RC-X haloalkane within the active site of a haloalkane dehalogenase (DmmarA numeration). The geometric limits considered are (Hur *et al.*, 2003): $d_{O\text{-}C} \leq 3.41$ Å, $\alpha_{O\text{-}C\text{-}X} \geq 157$ °, $d_{X\text{-}H} \leq 3.0$ Å.

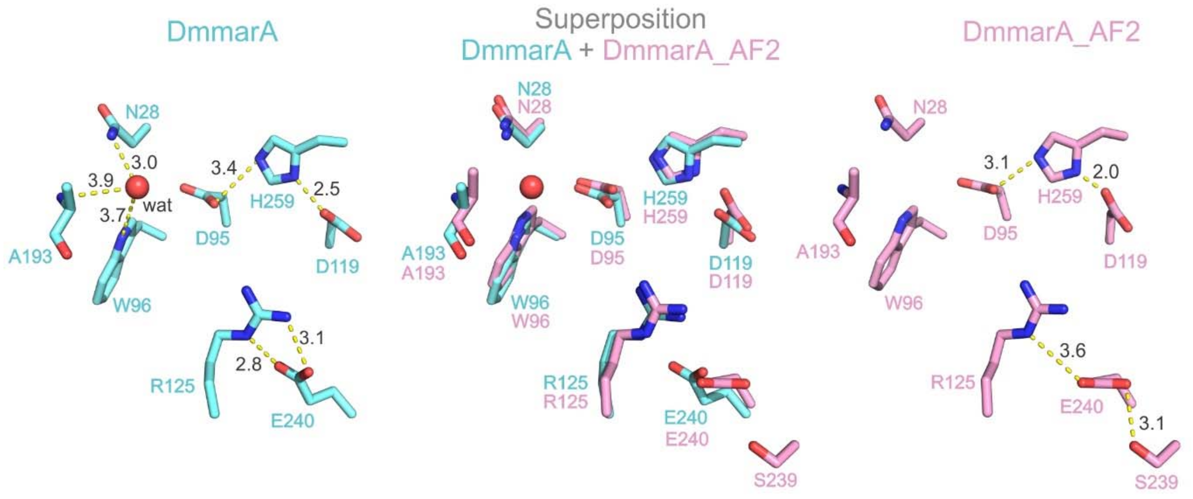

Figure S3 Active site superposition of DmmarA crystallographic structure with the structure predicted by Alphafold2 (DmmarA_AF2). The left panel depicts the active site of DmmarA, residues are displayed as blue sticks, hydrogen bonds between residues and with water are shown as yellow dashed lines, water is visualised as a red sphere. The middle panel displays the superposition of active sites of DmmarA (blue) and DmmarA predicted by Alphafold2 (pink). The right panel shows the DmmarA active site predicted by Alphafold2, the residues are displayed as pink sticks, bonds are shown as yellow dashed lines.

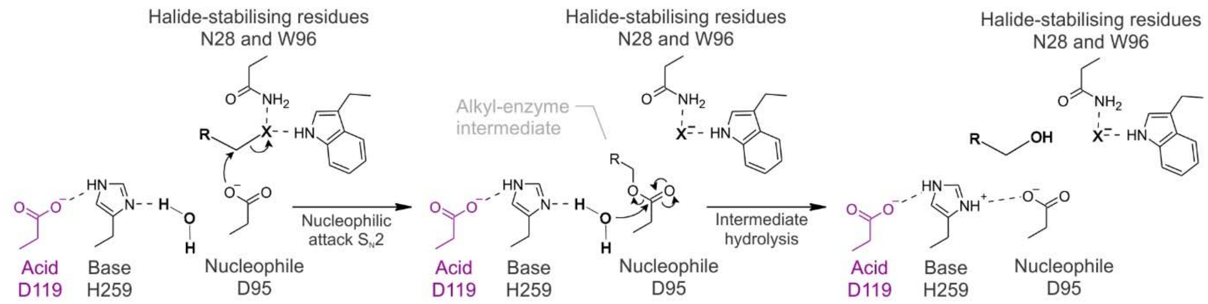

Figure S4 DmmarA haloalkane dehalogenase reaction scheme. The catalytic acid D119 unique compared to HLD-II subfamily is highlighted in purple. Depicted according to (Verschueren *et al.*, 1993) and (Koudelakova *et al.*, 2013).

References

Hur, S., Kahn, K. & Bruice, T. C. (2003). *Proceedings of the National Academy of Sciences* **100**, 2215–2219.

Koudelakova, T., Bidmanova, S., Dvorak, P., Pavelka, A., Chaloupkova, R., Prokop, Z. & Damborsky, J. (2013). *Biotechnology Journal* **8**, 32–45.

Liskova, V., Stepankova, V., Bednar, D., Brezovsky, J., Prokop, Z., Chaloupkova, R. & Damborsky, J. (2017). *Angew. Chem. Int. Ed.* **56**, 4719–4723.

Vasina, M., Vanacek, P., Hon, J., Kovar, D., Faldynova, H., Kunka, A., Buryska, T., Badenhorst, C. P. S., Mazurenko, S., Bednar, D., Stavrakis, S., Bornscheuer, U. T., deMello, A., Damborsky, J. & Prokop, Z. (2022). *Chem Catalysis* **2**, 2704–2725.

Verschueren, K. H. G., Seljée, F., Rozeboom, H. J., Kalk, K. H. & Dijkstra, B. W. (1993). *Nature* **363**, 693–698.
